# Novel VN/C nanocomposites as methanol-tolerant oxygen reduction electrocatalyst in alkaline electrolyte

**DOI:** 10.1038/srep11351

**Published:** 2015-06-23

**Authors:** K. Huang, K. Bi, C. Liang, S. Lin, R. Zhang, W. J. Wang, H. L. Tang, M. Lei

**Affiliations:** 1 State Key Laboratory of Information Photonics and Optical Communications & School of Science, Beijing University of Posts and Telecommunications, Beijing 100876, China; 2 Beijing National Laboratory for Condensed Matter Physics, Institute of Physics, Chinese Academy of Sciences, Beijing 100190, China; 3 State Key Laboratory of Advanced Technology for Materials Synthesis and Processing, Wuhan University of Technology, Wuhan 430070, PR China

## Abstract

A novel VN/C nanostructure consisting of VN nanoparticles and graphite-dominant carbon layers is synthesized by nitridation of V_2_O_5_ using melamine as reductant under inert atmosphere. High crystalline VN nanoparticles are observed to be uniformly distributed in carbon layers with an average size of *ca*13.45 nm. Moreover, the electrocatalytic performance of VN/C towards oxygen reduction reaction (ORR) in alkaline electrolyte is fascinating. The results show that VN/C has a considerable ORR activity, including a 75 percent value of the diffusion-limited current density and a 0.11 V smaller value about the onset potential with respect to Pt/C catalyst. Moreover, the excellent methanol-tolerance performance of VN/C has also been verified with 3 M methanol. Combined with the competitive prices, this VN/C nanocomposite can serve as an appropriate non-precious methanol-tolerant ORR catalyst for alkaline fuel cells.

As is well known to all, ion exchange membrane fuel cells or polymer electrolyte membrane fuel cell (PEMFC) have been extensively regarded as one of the most attractive high-efficient energy conversion devices in standby power, portable and vehicular applications^1^,^2^,^3^,^4^,^5^,^6^,^7^. However, due to the sluggish kinetics of the cathodic oxygen reduction reaction (ORR) especially in acid electrolyte, ORR performance restricts the efficiency of a fuel cell system in essential. To accelerate the ORR process, Pt-based catalysts as the state-of-the-art electrocatalysts have been extensively used and highly desired, which not only raise the cost but also suffer from CO or methanol poisoning^8^,^9^,^10^. Considering the superior kinetics of the ORR in alkaline solution to that in acidic media and a much wider range of less expensive but stable materials as efficient ORR catalysts in alkaline solution^11^,^12^, it is believed that replacing precious and nondurable Pt catalysts with cheap materials in alkaline fuel cell systems is a key issue for the commercialization of fuel cells.

Meanwhile, the appearance and development of anion exchange membranes in recent years have overcome the serious CO_2_-poisoning problem to KOH electrolyte which will reduce the ionic conductivity of the electrolyte and block the pores in the electrode^13^. There has been a growing interest in alkaline direct methanol fuel cell (ADMFC) due to the simplicity of the system and the adaptability of the liquid fuel^14^. However, besides the serious dependence on precious Pt catalyst for methanol oxidation and oxygen reduction reactions, the crossover of methanol from anode to cathode through the membrane will also reduce and poison the cathodic reduction process leading to an inferior performance of ADMFC^15^. Thus, the exploitation of non-precious methanol-tolerant oxygen reduction reaction (ORR) catalysts have become an essential issue. At present, carbon-supported transition metal/nitrogen (M-N_x_/C) materials, non-precious metal oxides, carbides, nitrides, oxynitrides, and carbonitrides have turned out to be promising inexpensive catalysts towards ORR for fuel cells^16^,^17^,^18^,^19^,^20^,^21^. Given the abundance in sources, fairly good activity and relatively high chemical stability for oxygen reduction, transition metal nitrides (TMNs) can be chosen to be appropriate candidates as non-precious methanol-tolerant ORR catalysts. What’s more, a contraction of the metal d-band can result in a greater density of states (DOS) near the Fermi level in comparison with the parent metal during the formation of TMNs. And the redistributions of DOS in TMNs further gives rise to their attractive catalytic activities similar to those of noble metals^22^. For example, it was found that carbon supported tungsten nitride (W_2_N/C) exhibited significant ORR stability after 100 hours of operation in a typical single PEM fuel cell setup, although with a relatively low onset potential of 0.6 V vs. RHE^23^. Molybdenum nitride materials supported on carbon black (MoN/C) were also investigated as ORR electrocatalysts by this group. It was found that MoN/C electrode displayed an open circuit potential of over 0.7 V in a single fuel cell setup as well as a notably stability within 60 hours^24^. It was further demonstrated that the effective reduction of MoN/C catalysts can be attributed to the hexagonal MoN structure formed during the synthesis^25^. Recently, VN-based materials with some similar characteristics to those of aforementioned transition-metal nitrides such as corrosion resistance, high melting points and good electrical conductivity, have been researched in the applications of dye-sensitized solar cells, supercapacitors and lithium-ion batteries^26^,^27^,^28^, whereas few works with regards to ORR of carbon supported vanadium nitride (VN/C) were reported^29^.

Herein, we report a novel VN/C nanocomposite with a uniform distribution of VN nanoparticles on carbon layers as methanol-tolerant oxygen reduction electrocatalyst for alkaline fuel cell. The methanol-tolerant ORR performance of VN/C electrode was investigated by cyclic voltammetry (CV), rotating disk electrode (RDE) and chronoamperometry test with or without 3 M methanol. Benefiting from the unique structure and components of VN/C, this novel nanocomposite exhibits a considerable ORR activity with good stability and excellent methanol-tolerant performance.

## Results

**Figure 1:**
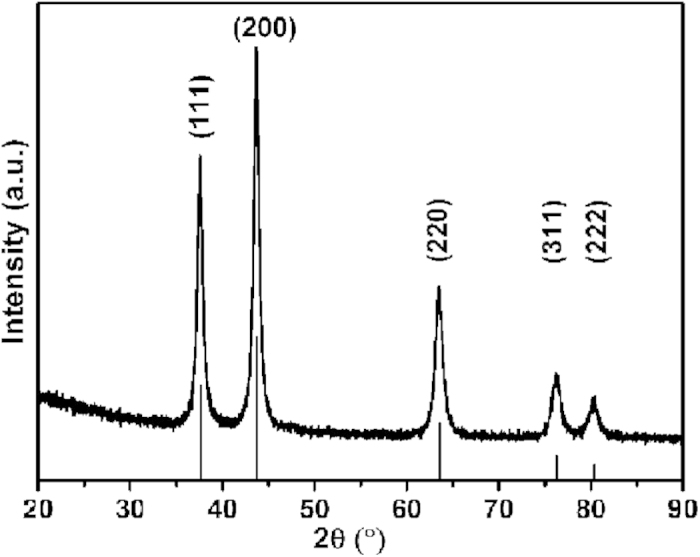
XRD Patterns of VN/C with the calculated pattern (JCPDS No. 35-0768).

The XRD pattern of as-synthesized powder is taken for structural characterization. As shown in Fig. 1, a series of diffraction peaks at 2*θ* values of 37.6, 43.7, 63.5, 76.3 and 80.2° can be observed, corresponding to the typical stoichiometric face-centered (fcc) VN structure (JCPDS No. 35–0768) with a lattice parameter of 0.414 nm. No other obvious diffraction peaks corresponding to vanadium oxide can be found in the pattern, suggesting the high purity and good crystallization of VN sample. Furthermore, the average crystallite size of VN crystals calculated by Scherrer equation is 13.2 nm from all the five crystal planes.

**Figure 2:**
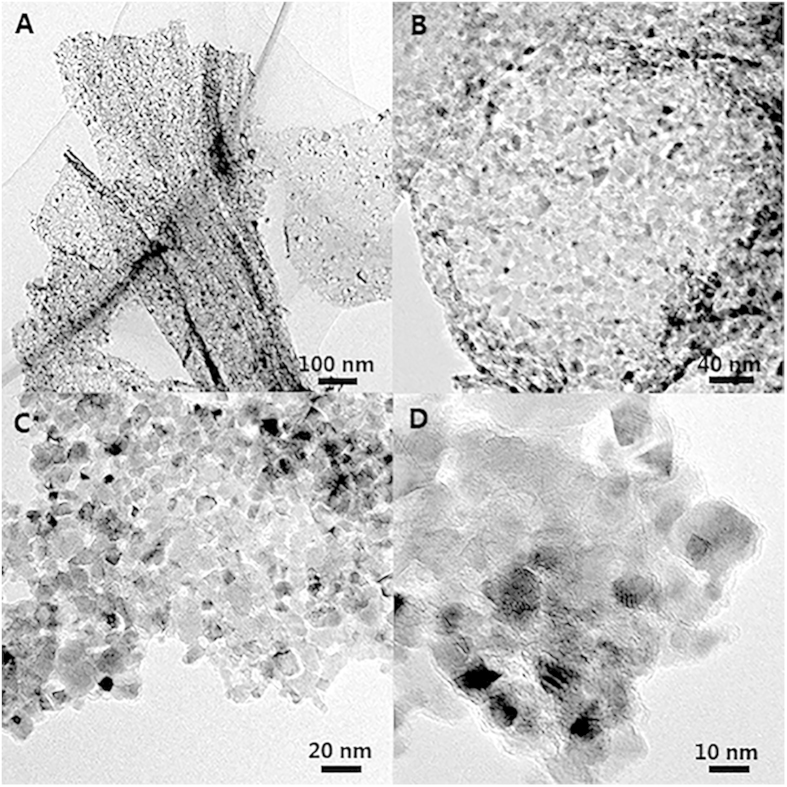
TEM images of VN/C at different magnifications.

**Figure 3:**
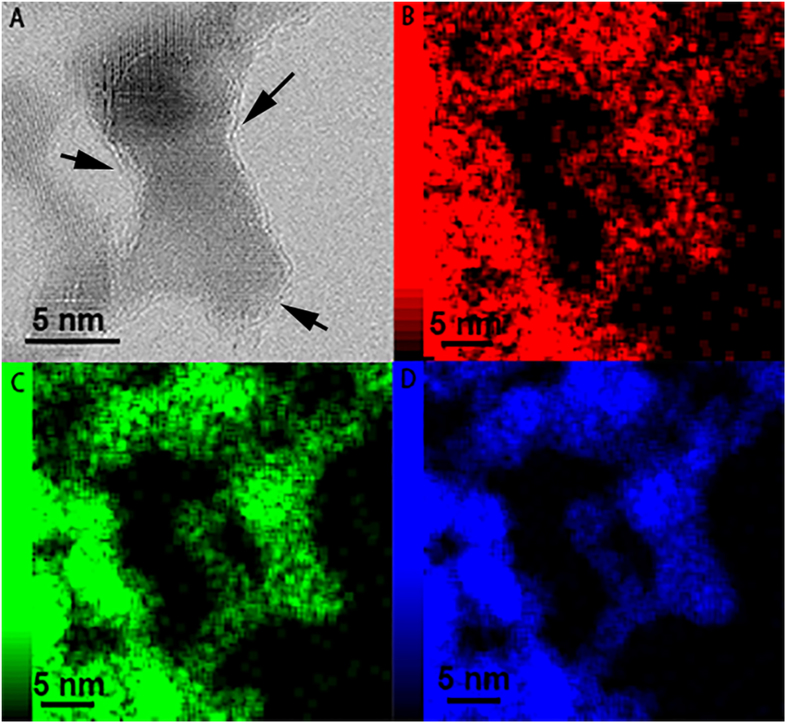
HR-TEM image (**A**) and element mapping spectra C (**B**), N (**C**) and V (**D**) of VN/C. The arrows indicate the surface carbon layers on VN Nanoparticles.

In order to reveal the physical morphology of VN visually, TEM images at different magnifications and element mapping spectra were displayed in Fig. 2 and Fig. 3. Surprisingly, as-prepared VN sample is found out to be a kind of composite consisting of nanoparticles and surrounding layers which is very similar to the structure of metal oxides nanoparticles supported on graphene sheets. Although the average size of nanoparticles (*ca* 13.45 nm, see Fig. S1) matches well with the results calculated from XRD analysis, the existence of surrounding layers seems to be contradictory. Considering the synthesis condition and process: the pyrolysis of melamine will release NH_3_ and some chemically reactive hydrogen-, carbon-, and nitrogen-containing atomic species at higher temperatures^30^, it is believed that the surrounding layers are composed of carbon layers which may grow by a self-catalytic process by VN nanocrystals as reported by Yao *et al*^31^ and exhibit no obvious diffraction peaks due to the destroyed regular stacks of carbon layers^32^.

Energy dispersive X-ray spectrum (EDS) in Figure S2 and Raman spectroscopy (Figure S3) were further recorded for the componential analysis of VN-based nanocomposite. Only three strong vanadium peaks at 4.96, 5.43, 0.51 keV, nitrogen peak at 0.39 keV and carbon peak at 0.27 keV can be observed^33^. Moreover, the quantitative analysis indicates that the atomic percent of vanadium, nitrogen and carbon is at a ratio of 35.27: 34.36: 30.37, demonstrating that as-prepared sample can be identified as VN/C nanocomposites. Meanwhile, two Raman peaks at 1401.4 and 1586.2 cm^−1^ are observed in Raman spectra, which can be attributed to the disorder induced D-band and G-band of crystalline graphite, respectively. The ratio of G-band with respect to D-band I_G_/I_D_ obtained from the smooth line is 1.34, indicating that the carbon layer can be attributed to graphitic domains^34^,^35^.

**Figure 4:**
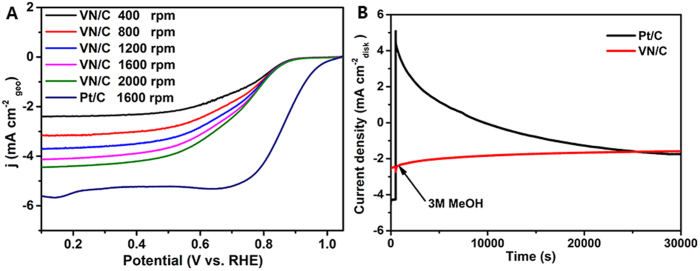
ORR activity (A) and the chronoamperometric response upon adding 3 M MeOH (B) of VN/C catalyst and commercial Pt/C catalyst.

Figure 4 shows the ORR polarization curves and chronoamperometric responses upon adding 3 M MeOH of VN/C electrode with respect to commercial Pt/C electrode. Clearly, VN/C catalyst exhibits a very similar tendency (under a combined kinetic-diffusion control of charge transfer and mass transport) with commercial Pt/C catalyst in O_2_-sataured 0.1 M KOH electrolyte. The ORR onset potential of VN/C electrode is 0.87 V Vs. RHE (0.11 V smaller than Pt/C electrode), the half-wave potentials of VN/C and Pt/C electrodes are 0.73 and 0.85 V respectively. Meanwhile, the diffusion-limited current density of VN/C electrode is 4.12 mA cm^−2^ at 1600 rpm, almost 80 percent of that of Pt/C electrode. Figure S4 also indicates the stability of VN/C nanocomposites overmatches that of commercial Pt/C catalysts with a more gradual and limited degeneration. In addition, VN/C electrode shows negligible change in its ORR current density upon adding 3 M methanol at 500 s, while an instantaneous current jump due to the initiation of methanol oxidation reaction (MOR) is observed for Pt/C electrode^36^. The superior methanol tolerance and considerable ORR activity of VN/C illuminate us its powerful competitiveness of being an alternative for non-precious methanol-tolerant ORR catalyst.

## Discussion

**Figure 5:**
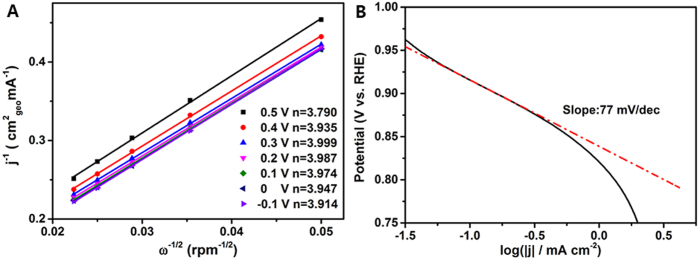
Koutechy-Levich (K-L) plots (**A**) at different electrode potentials for VN/C and Tafel slope (**B**) of VN/C derived by the mass-transport correction of RDE data at 1600 rpm.

It is well known to all that there are two major pathways for the reduction of oxygen in alkaline aqueous solution: direct 4-electron pathway to “hydroxyl ion” and 2-electron pathway with “peroxide” as the reduction product. According to Koutechy-Levich theory, the electron transfer number in ORR can be calculated using equations as follows:
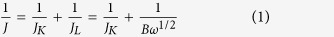

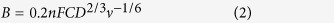
where *J* and *J*_*K*_ are the measured and kinetic-limiting current densities, ***ω*** is the rotation speed (rpm), *n* is the transferred electron number, *F* is the Faraday constant (*F* = 96485 C mol^−1^), *C* is the concentration of O_2_ in 0.1 M KOH solution (*C* = 1.2 × 10^−6^ mol cm^−3^), *D* is the diffusion coefficient of O_2_ (*D* = 1.9 × 10^−5^ cm^2^ s^−1^), ***V*** is the kinematic viscosity (***V*** = 0.01 cm^2^ s^−1^) referring to the previous reports^37^,^38^. Based on the Rotating-disk voltammograms of VN/C electrode in O_2_-saturated 0.1 M KOH solution from 400 to 2000 rpm in Fig. 4A, the average number of electrons transferred per oxygen molecule involved in VN/C catalyst for ORR is 3.95 which suggests a direct 4-electron reduction process (Fig. 5A). Moreover, on the basis of Tafel equations:
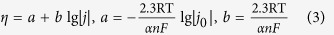


the Tafel slope of VN/C catalyst derived by the mass-transport correction of RDE data at 1600 rpm is 77 mV decade^−1^, thus the exchange current density j_0_ can be calculated to be about 5.66 × 10^−8^ mA cm^−2^, which is very close to the values of Pt/C catalyst and M-N_x_/C catalysts^39^. In a view of analysis above, VN/C can be regarded as an inexpensive alternative ORR catalyst with considerable catalytic activity.

**Figure 6:**
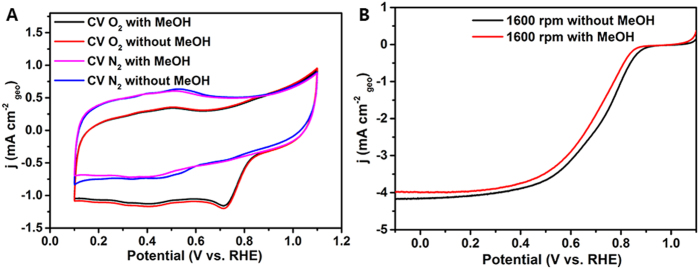
Methanol tolerant measurements of VN/C: Cyclic voltammetry curves (**A**) of VN/C as ORR catalysts with or without 3 M MeOH in N_2_-saturated and O_2_− saturated 0.1 M KOH and LSV curves (**B**) of VN/C electrode at 1600 rpm with or without 3 M MeOH.

Considering the methanol crossover effect for practical application in ADMFC and our ultimate goal of developing non-precious methanol-tolerant ORR catalysts, cyclic voltammograms and linear sweep voltammograms were further recorded for VN/C electrode in 0.1 M KOH solution with 3 M MeOH. As shown in Fig. 6A, no significant oxidation or reduction current peak is observed when methanol was presented in the CV curves recorded from both N_2_ and O_2_ saturated solution at a scan rate of 50 mV s^−1^, demonstrating that the VN/C is chemically stable in methanol. Moreover, the ORR onset potential, half-wave potential and diffusion-limited current density of VN/C electrode in O_2_ saturated 0.1 M KOH with 3 M MeOH are 0.865 V, 0.695 V and 3.987 mA cm^−2^ (Fig. 6B), which is very close to the values without 3 M MeOH. The different responses reveal that the remarkably superior methanol tolerance and high catalytic selectivity against methanol of VN/C to commercial Pt/C as the ORR catalyst should be attributed to the inertness of VN/C toward methanol oxidation. In addition, the methanol-tolerant performance of this newly developed VN/C catalyst is superior to other non-noble metal methanol-tolerant ORR catalysts, such as carbon-encapsulated cobalt-tungsten carbide catalyst^40^, carbon-supported MoN catalyst^41^ and our previous work of AlN nanowires catalyst^36^.

**Figure 7:**
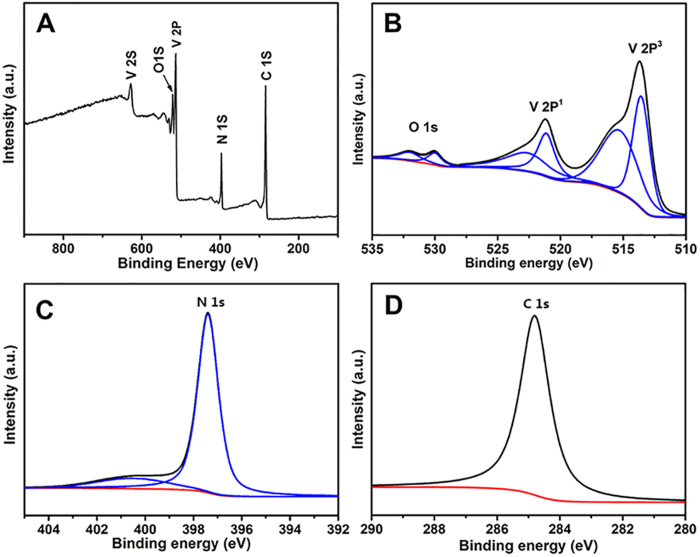
XPS spectra of the surface of VN/C nanocomposite.

It is because that the ORR process is a function of the consumption of oxygen at the surface of a catalyst, the surface chemical states are closely related to its activity and durability. XPS spectra of VN/C catalyst are further studied to feature the surface of VN/C nanocomposite in Fig. 7, it can be seen that the surface of VN/C mainly consists of V, N, O, and C species elements from the survey spectrum. The appearance of O species seems to be unanticipated as no O element was detected by EDS analysis, the most possible reasons are that O element is relative limited and mainly exists in the surface of VN/C sample which is overlapped by high-intensity V peak and thus beyond the detection precision of EDS^42^. Figure 7B shows the high-resolution XPS spectra of O 1 s and V 2p, indicating the presence of an oxide layer on the surface of VN. The peaks at 532.2 and 529.9 eV of O1s can be attributed to lattice oxygen in a metal oxide and adsorbed hydroxyl oxygen (OH^−^) on the surface of VN/C, respectively^43^,^44^,^45^. The V (2p_3/2_) and V (2p_1/2_) peaks centered at 513.6 and 521.1 eV belong to vanadium in the VN crystalline while the other two peaks at 515.3 and 522.6 eV can be assigned to oxidation states of vanadium in surface oxynitrides VO_x_N_y_ owing to the passivation of VN in air as a protective oxide layer of the surface to avoid the strong oxidation^45^,^46^,^47^. Figure 7C shows the N 1 s spectrum of VN/C, the peak at 397.4 eV is the typical characteristic of metal nitride (VN in this situation) while the peak at 400.2 eV can be generally attributed to the adsorbed NH_3_ at the surface of VN/C deriving from the decomposition of melamine at high temperature^30^,^48^,^49^. Moreover, the C1s profile is composed of a single symmetric peak as shown in Fig. 7D, which means that the C element in VN/C is just carbon and no carbides or carbonitrides exists in VN/C. Thus, the core-shell structure as well as the nanocrystallization of VN particles is believed to be favorable to enhance ORR electrocatalytic activity similar to the report by Bao and his co-workers^50^, while the VO_x_N_y_ layer and graphitic layers on the surface of VN nanoparticles benefit the tolerance of methanol.

In conclusion, a novel VN/C catalyst elelctrochemically stable in alkaline conditions with or without methanol for ORR is successfully synthesized by the reduction of V_2_O_5_ using melamine as single nitridizing reagent under N_2_. It is found that VN/C exhibits a considerable ORR activity with an onset potential at about 0.87 V vs. RHE, a high cathodic diffusion-limited current density of 4.12 mA cm^−2^ and an exchange current density of about 5.66 × 10^−8^ mA cm^−2^. No obvious degradation of the ORR performance upon the methanol addition in the electrolyte is observed in the cyclic voltammetry curves (CVs), linear sweep voltammograms (LSVs) and i-t curves. All electrochemical results demonstrated the VN/C nanocomposite catalyst is a promising non-precious metal methanol-tolerant candidate as ORR electrocatalyst for alkaline fuel cells.

## Methods

### Preparation of VN/C nanocomposite

All starting materials are of analytical pure grade and are purchased from commercial sources. In the typical synthesis, 80 mmol melamine and 20 mmol V_2_O_5_ were firstly mixed together, pressed to a pellet and further put in an alumina boat. Then, the alumina boat was placed in the center of a horizontal tubular furnace with two open-end straight alumina tube. After flushed with nitrogen atmosphere to remove the remaining air in the alumina tube, the furnace temperature was heated to 900 °C and kept at the peak temperature for 6 hours under N_2_ flow at 150 sccm. After the furnace temperature was cooled to the room temperature in the flow of N_2_ atmosphere, the black product was obtained in the alumina boat.

### Structure analysis of the VN/C nanocomposite

Morphology and microstructure of the samples were characterized by powder X-ray diffractometry (XRD, X’pert PRO, Panalytical) using Cu Kα as radiation source, transmission electron microscopy (TEM, ARM200F, JEM) equipped with energy-dispersive X-ray spectroscopy (EDS) and Raman spectroscopy (LabRAM Aramis, Horiba Jobin Yivon). XPS measurements were carried out on an ESCALAB 250Xi spectrometer by Thermo Scientific with C 1 s peak at 284.8 eV as an internal standard.

### Electrochemical measurement of the VN/C nanocomposite

Electrochemical characterizations were performed on a CHI660E electrochemical workstation with a three-electrode system consist of a glassy carbon electrode of 5 mm in diameter as the working electrode, a Pt foil as the counter electrode and an Hg/HgO electrode as the reference electrode. All potential values were calibrated with respect to reversible hydrogen electrode (RHE) by E (RHE) = E (Hg/HgO) + 0.92 V. For electrode preparation, VN/C and commercial Pt/C (20 wt.% Pt on Vulcan XC-72, Johnson Matthey) were dispersed in the mixture of 700 μl of ultrapure water, 300 μl of iso-propanol and 20 μl of Nafion solution (5.0 wt.%) by sonication for 30 min in ice-bath to form the homogeneous ink, then 20 μl of the catalysts ink was loaded onto the glassy carbon electrode and dried under ambient temperature with a catalyst loading of 0.5 mg cm^−2^. CV measurements were performed from 0.1 to 1.1 V at a scan rate of 50 mv s^−1^ in N_2_− and O_2_− saturated 0.1 M KOH solution with or without 3 M MeOH, respectively. Rotating disk electrode (RDE) measurements were conducted at different rotating speeds from 400 to 2000 rpm in O_2_− saturated 0.1 M KOH solution at a scan rate of 5 mv s^−1^ and chronoamperometry was carried on a constant voltage of 0.65 V in O_2_−saturated 0.1 M KOH solution with adding 3 M methanol at 500 s in succession.

## Additional Information

**How to cite this article**: Huang, K. *et al*. Novel VN/C nanocomposites as methanol-tolerant oxygen reduction electrocatalyst in alkaline electrolyte. *Sci. Rep*. **5**, 11351; doi: 10.1038/srep11351 (2015).

## Supplementary Material

Supplementary Information
