# Supplementary material for: Novel VN/C nanocomposites as methanol-tolerant oxygen reduction electrocatalyst in alkaline electrolyte
Source: Sci Rep. 2015 Jun 23;5:11351. doi: 10.1038/srep11351 (PMC4477409; doi:10.1038/srep11351)
Supplement: Supplementary Information [file srep11351-s1.doc]

**Support Information**

**Novel VN/C nanocomposites as methanol-tolerant oxygen reduction electrocatalyst in alkaline electrolyte**

K. Huang^1^, K. Bi^1^, C. Liang^1^, S. Lin^1^, R. Zhang^1^, W.J. Wang^2^, H.L. Tang^3^, M. Lei^1^

*^1^State Key Laboratory of Information Photonics and Optical Communications &School of Science, Beijing University of Posts and Telecommunications, Beijing 100876, China.*

*^2^Beijing National Laboratory for Condensed Matter Physics, Institute of Physics, Chinese Academy of Sciences, Beijing 100190, China.*

*^3^State Key Laboratory of Advanced Technology for Materials Synthesis and Processing, Wuhan University of Technology, Wuhan 430070, PR China.*

*Correspondence and requests for materials should be addressed to M.L. (minglei@bupt.edu.cn) or H.L.T. (thln@whut.edu.cn) or W.J.W.(*[*wjwang@iphy.ac.cn*](mailto:wjwang@iphy.ac.cn)*).*


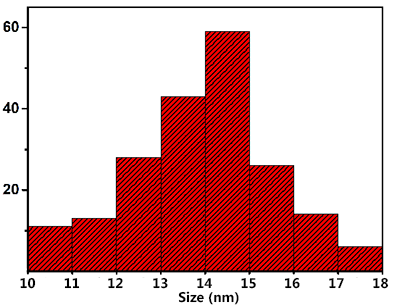


**Figure S1.** The size distribution of VN Nanoparticles based on counting 200 particles.


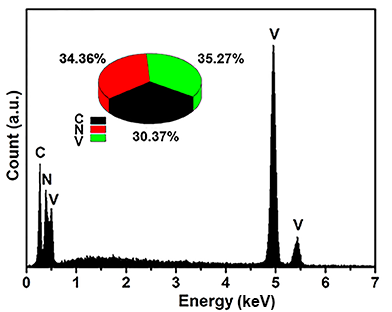


**Figure S2.** EDS spectrum showing the chemical species of VN/C.

**
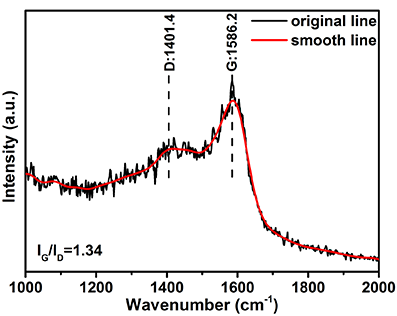
**

**Figrue S3.** Raman spectrum of carbon in VN/C.

**
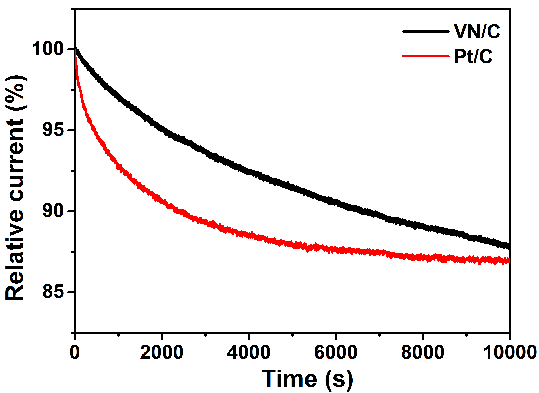
**

**Figrue S4.** The i-t curves for VN/C and Pt/C catalysts at 1600 rpm at 0.65 V in O_2_-saturated 0.1 M KOH solution.
